# E-learning and E-modules in medical education—A SOAR analysis using perception of undergraduate students

**DOI:** 10.1371/journal.pone.0284882

**Published:** 2023-05-19

**Authors:** Archana Prabu Kumar, Abirami Omprakash, Prabu Kumar Chokkalingam Mani, Maheshkumar Kuppusamy, Doaa Wael, B. W. C. Sathiyasekaran, P. V. Vijayaraghavan, Padmavathi Ramasamy

**Affiliations:** 1 Medical Education Department, College of Medicine and Medical Sciences, Arabian Gulf University, Manama, Bahrain; 2 Department of Physiology, Sri Ramachandra Medical College and Research Institute, Sri Ramachandra Institute of Higher Education and Research, Porur, Chennai, Tamil Nadu, India; 3 Department of Biochemistry, Sri Ramachandra Medical College and Research Institute, Sri Ramachandra Institute of Higher Education and Research, Porur, Chennai, Tamil Nadu, India; 4 Department of Physiology, Government Yoga and Naturopathy Medical College and Hospital, Chennai, Tamil Nadu, India; 5 Faculty of Medicine, Ain Shams University, Cairo, Egypt; 6 Department of Orthopaedics, Sri Ramachandra Medical College and Research Institute, Sri Ramachandra Institute of Higher Education and Research, Porur, Chennai, Tamil Nadu, India; 7 Sree Balaji Medical College and Hospital (SBMCH), Balaji Institute of Higher Education and Research (BIHER), Chennai, Tamil Nadu, India; Prince Sattam bin Abdulaziz University Faculty of Medicine, SAUDI ARABIA

## Abstract

**Background:**

Application of e-learning and e-modules in medical education has been shown to have a positive impact on learning outcomes among all types of learners, across diverse educational settings. Despite its benefits, e-learning and e-modules has not yet reached its full potential in medical education in India. Objective of this study is to evaluate the perception of undergraduate students regarding e-learning and e-modules using an appreciative inquiry tool SOAR (Strengths, Opportunities, Aspirations, Results) analysis, and to identify the barriers and challenges for the same.

**Methods:**

This longitudinal study was conducted among participants from three consecutive batches (n = 250 x 3) of first-year medical students and two consecutive batches (n = 100 x 2) of first-year dental students. The sample was selected using a purposive sampling method. Two structured and validated questionnaires were developed for this study based on the modified Zhou’s Mixed Methods Model; the ‘Knowledge, Attitude and Practice’ Questionnaire (KAPQ) on e-learning and the feedback questionnaire (FBQ) on e-modules. The questionnaires were administered via MOODLE / hard copy, before and after the implementation of e-modules, respectively. Identified strengths, potential opportunities, probable aspirations and likely results for e-learning and e-modules were tabulated based on the qualitative analysis of perceptions of large number students sampled across three years.

**Results:**

Six hundred and ninety students returned both questionnaires representing a response rate of 76.6%. Nine themes were identified in the “Strengths” domain as follows: Regular Update of Knowledge, Innovative Learning, Availability, Knowledge Sharing, Abundance of Information, Accessibility, Source of Knowledge, Creativity, and Increased Engagement. Eleven themes were identified in the “Opportunities” domain as follows: Clinical Skills training, Timesaving, Flexibility, Creativity, Increased engagement, Standardized content, Capacity building for students, Capacity building for faculty, Skills training, and Self-assessment. Thirteen themes were identified under the “Aspirations” domain with the three key themes being “maintaining and building on current strengths”, “increasing potential opportunities”, and “addressing the barriers and challenges identified in the responses to the KAPQ and FBQ questionnaire”. Four themes identified for ‘Barriers’ were eye strain, distractions, preference for conventional methodologies, and internet connectivity.

**Conclusions:**

The findings of this qualitative study are based on the responses received from first-year medical and dental students of a Private University in Chennai, India. In this population of students, implementation of e-learning as blended learning using structured and interactive e-modules may provide more engagement during learning as well as support self-directed learning (SDL) directly or indirectly. Adoption of blended learning with e-modules as an integral part of curriculum planning may be beneficial for the achievement of Competency-Based Medical Education (CBME) goals in India.

## Introduction

Training in medical education has been vastly influenced by technology and the e-revolution of the past 20 years [1, 2]. E-learning, use of the internet and other related tools for learning, has become mainstream across the globe. Going beyond its broad characterization as online learning, Ellaway and Masters (2008) defined e-learning as *“a pedagogical approach that typically aspires to be flexible*, *engaging and learner-centered; one that encourages interaction (staff-staff*, *staff-student*, *student-student)*, *and collaboration and communication*, *often asynchronously (though not exclusively so)”* [3]. E-learning is projected to continue to remain a key method of training delivery in health professional education (HPE) [4, 5]. The role of e-learning in achieving the educational goals of HPE, especially in developing and under-developed countries has also been recognized by the WHO [6].

E-learning may be made more engaging and tailored to the delivery of formal education goals when supplemented with curricula-focused electronic lessons. E-modules are electronic lessons focusing on important learning concepts, built with a combination of teaching and assessment tools including text, images, audio, video, and gaming elements [7].

Application of e-learning and e-modules in medical education has been shown to have a positive impact on learning outcomes among all types of learners, across diverse educational settings [8, 9].

Globally, many studies have documented the perceptions of medical students about e-learning. The overall consensus is that the acceptance rate of e-learning among medical students range from moderate to good. Many students have also indicated that additional digital training, superior designing of e-courses with enhanced interaction, and blending of traditional teaching with online methods would prove more beneficial [1, 10, 11]. However, in some countries, students did not favor e-learning [12]. These findings highlight the need to devise a strategic blueprint for e-learning implementation that meets the needs of students in a manner that is specific to each region.

Despite its benefits, e-learning has not yet reached its full potential in India. The Medical Council of India / National Medical Commission (MCI/NMC) advocated for innovative concepts including self-directed learning (SDL) and active teaching-learning methods as part of pedagogical strategies to deliver Competency-Based Medical Education (CBME) [13–15]. The capability of e-learning and digital tools to support SDL in formal education is well-established [16]. When used appropriately, e-modules are shown to persuade students toward lifelong learning [17]. It is, therefore, imperative to develop and implement an evidence-based road map for improving e-learning in medical education within the Indian context based on a careful assessment of the strengths, possible impact, and potential opportunities of this pedagogical approach.

SOAR (Strengths, Opportunities, Aspirations, Results) analysis is an appreciative inquiry tool that is uniquely tailored to enable strategic planning around well-defined goals [18]. SOAR analysis differs from the well-known SWOT (strengths, weaknesses, opportunities, and threats) analysis within two dimensions; it focuses on the future prospects and results from a subject of interest while SWOT aims at inherent weaknesses and perceived threats [19]. SOAR is guided by ‘achieving the good’ rather than ‘avoiding an error’. SOAR is also driven by action, plan, and results [20]. Literature search shows lacunae in SOAR analysis specific to the Indian context that takes into account knowledge, attitude, and practice of e-learning among undergraduate medical students [14, 15]. Hence, the present study was planned to study the perception of undergraduate medical students regarding e-modules during pre-covid times. Since e-learning is projected to remain an integral part of medical education, the results can be used to improve e-module preparation and delivery for HPE as an integral curricula strategy.

### Objective

To evaluate the perception of undergraduate medical students regarding e-learning and e-modules using structured and validated questionnaires and SOAR analysis. A secondary objective of the study is to identify the barriers and challenges for the implementation of e-modules in undergraduate medical curricula in India, as perceived by undergraduate medical students.

## Methods

Two structured and validated questionnaires were developed for this study based on the modified Zhou’s Mixed Methods Model; the ‘Knowledge, Attitude and Practice’ Questionnaire (KAPQ) on e-learning and the feedback questionnaire (FBQ) on e-modules.

### Item generation

Exhaustive literature review was carried out by authors to find existing validated questionnaires that were used in similar research settings. In addition, several informal focus group discussions were held with the head of the e-learning unit, phase one curriculum coordinator, educational experts and student representatives under the guidance of the Dean—Education, and Associate Dean of Education. The deliberations during the meetings were transcribed verbatim into a word document by secretaries. Experts in qualitative research performed thematic analysis on the captured data, resulting in the generation of themes that became the items on the KAP questionnaire (KAPQ) and feedback questionnaire (FBQ).

### Data collection tools

The KAPQ comprised of 10 domains namely, general information (11 items), availability of computers & internet connectivity (6 items), purpose of using internet (6 items), self-reported confidence levels (10 items), preferred resources used for studying (6 items), preferred components of e-module (8 items), reasons for preferring e-learning (7 items), limitations of e-learning (11 items), and applications of e-learning in education (7 items). These were followed by three open-ended questions on major advantages, major disadvantages and the biggest challenge for e-learning in India [21]. The FBQ consisted of 14 items and four open-ended questions, on what students thought they ‘liked the most’, ‘did not like’, ‘could have been done better’, and ‘suggestions for the improvement of e-modules’. To facilitate better understanding of the questions, the KAPQ and FBQ were constructed in simple English and scored on a five-point Likert Scale [22]. Relevance of the content, level of complexity of the language, sequence of items from simple to complex, and technique of administration of the questionnaires were taken into account when considering valid responses to the KAPQ and FBQ.

### Validation

Face validity was carried out by three faculty who were not experts in e-learning.

To establish content validity, the questionnaires were examined by three internal and external experts based on the Lawshe method [23]. The experts belonged to a pool of faculty using e-learning for teaching and assessment, or medical educationists. Acceptance by five out of six experts was considered as a criterion for including the specified item in the KAPQ and FBQ. The validation exercises resulted in considerable modifications and improvement of the questionnaires.

### Participants and procedures

Ethics Committee clearance was obtained from Institutional Ethics Committee (IEC-NI/12/OCT/30/53) and written informed consent was obtained from all participants. The sample was selected based on the purposive sampling method. The data was collected from three consecutive batches (n = 250 x 3) of first-year MBBS students (2014–2017) and two consecutive batches (n = 100 x 2) of first-year BDS students (2015 to 2017). KAPQ and FBQ were administered to 900 students before and after implementation of e-modules respectively, through MOODLE / hard copy. 690 students returned both the questionnaires (response rate = 76.6%). The participant characteristics are shown in Table 1.

**Table 1:** Participant characteristics.

Characteristics	Distribution			
Male	272 (39%)			
Female	418 (61%)			
Age Group	All participants belong to age group 17–19 (*n* = 690)			
Medical	554 (80%)			
Dental	136 (20%)			
Year of study	All participants belong to first year (*n* = 690)			
Batch wise breakdown	*Medical*	*Dental*	*Male*	*Female*
*2014–2015*	196	0	66	130
*2015–2016*	169	73	102	140
*2016–2017*	189	63	104	148

### Data analysis

**Fig 1:**
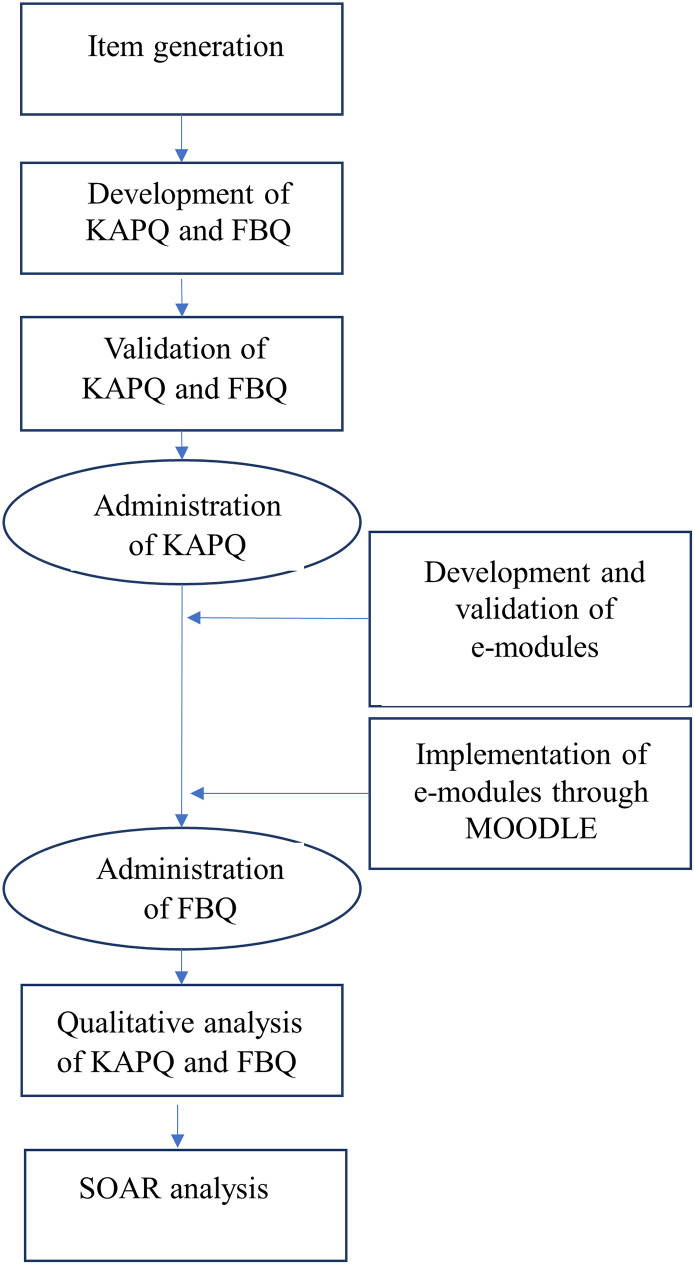
Flowchart showing the overview of methodology.

Collation of responses and descriptive data analysis was done across all the KAPQ and FBQ domains using SPSS version 17. The qualitative responses were then analyzed using thematic analysis [24]. A deductive approach was followed while interpreting the transcribed data and an inductive approach was utilized for recognizing patterns and generating themes [25]. The generated themes were examined carefully through a peer-review process to ensure internal similarity and external variability. SOAR analysis was performed on the findings. Fig 1 shows the overview of methodology. A pilot study was carried out for validating the questionnaire [26]. The quantitative responses of the present study were analyzed and published [27].

## Results

### Strengths

Nine themes were identified under this domain follows: regular update of knowledge, innovative learning, availability, knowledge sharing, abundance of information, accessibility, source of knowledge, creativity, and increased engagement. Of the nine “strengths:, the two predominant themes under this domain were ‘Accessibility’ and ‘Knowledge sharing’. E-modules are easily accessible; they are available even after the end of the lesson sessions and can be re-visited at any time. E-learning facilitates easy access to up-to-date knowledge in the medical field. Innovative learning and increased engagement were facilitated by the use of electronic tools such as videos and animations.

### Opportunities

Eleven “opportunities” were identified in the raw data as follows: Clinical Skills training, Time saving, Flexibility, Creativity, Increased engagement, Standardized content, Capacity building for students, Capacity building for faculty, Skills training, and Self-assessment. The main themes under this domain were ‘Creativity’ and ‘Increased engagement’. Creativity is an essential skill for medical workers that allows them to explain and convey clinical information in an effective manner to a recipient. Digital elements such as animations used in the e-modules were found to boost students’ creativity. Use of visual aids and videos made learning more engaging compared to traditional methods of teaching such as lectures and seminars.

### Aspirations

Thirteen themes emerged in the “aspirations” domain as follows: Dedicated IT personnel; Panel of educational experts for periodic content review; Increase students’ engagement (illustrative videos, 3D images, interactive flash games, etc.); Hire a digital designer to work with educational experts; Standardize the e-learning process across universities; Provide official faculty development program on IT and multimedia; "Hours spent” metric and progress bar; Interactive games and visuals for clinical skills development; Forum discussion, post-test and self-assessment tests with personal leaderboard display), Streamline student enrollment; E-modules marks to be part of formative/summative evaluation, and Continuous development (periodic student/faculty feedback on content, technology, delivery, accessibility, quality, and other aspects that assess the quality of the e-learning experience). Of these aspiration themes, three key themes: maintaining and building on current strengths, increasing potential opportunities, and addressing the barriers and challenges identified in the responses to the KAPQ questionnaire.

#### Maintaining and building on current strengths

Increasing students’ confidence and comfort level with IT-based learning are imperative. These goals can be achieved by providing a tutorial at the beginning of the semester to introduce the students to the e-learning platform and walk them through how to access their e-modules and post-tests. Providing a dedicated IT personnel will allow for consistency in website maintenance and rapid resolution of any technical issues that may arise.

#### Increasing potential opportunities

Students’ engagement and learning can be enhanced through the strategic use of illustrative videos and visual data in all e-learning modules. For example, interactive games and visuals can be introduced to guide students through technical and clinical procedures such as blood pressure measurement and minor surgery. The provision of discussion forums, post-test, and self-assessments may contribute to enhanced student learning.

Self-directed learning can be enhanced through the use of strategies such as “Hours spent" metric and “progress bar” that allows students to track how much time they spend learning online and how much they have completed so far.

Innovative techniques such as the introduction of a ‘download’ option that allows students to view pre-downloaded lectures and modules, while eliminating the need for a steady online connection in order to access lessons. Annual/periodic review of e-modules to be undertaken by a panel of educational experts assigned by the department head, to revise and update the information introduced in the e-modules as per approved educational curriculum and best-in-class/current evidence.

A list of resources such as websites that provide free-to-use visual aid material including 3D images geared towards medical content to be provided to teachers to support lesson creation.

A digital designer to be assigned to work closely with the lesson planning committee to create engaging e- module products based on the guidance provided by educational experts on the needed content.

Official faculty development programs geared towards the creation of effective online lessons, including technical details such as ensuring variability in voice tone to avoid monotoy, will support the effective delivery of e-learning.

Broader efforts to improve e-learning applications will include collaboration with other universities to exchange expertise and an attempt to standardize the e-learning process for medical education across universities.

#### Addressing barriers and challenges identified in the responses to the KAPQ questionnaire

All the barriers identified are mentioned under the ‘barriers’ section.

## Results

In line with the anticipated continuation of e-learning as an integral pedagogical approach in medical education, all upcoming student batches would be enrolled in e-modules.

The progress and attainment of identified aspirations will be measured primarily using students’ responses via periodic feedback questionnaires. Questionnaire domains would include content, technology, delivery, accessibility, and other aspects that indicate the quality of the e-learning experience.

The target goal for students’ confidence level in using IT-based learning tools is 99%.

A student satisfaction rate of 90% and above on the e-learning experience would indicate a successful e-learning model.

Periodic modifications may be made to the e-learning strategy based on feedback from students and faculty.

An increase in the total number of hours spent on the institution’s e-learning platform is a positive indicator of utilization of the platform. The minimum engagement target is 85% for time spent on the e-learning platform and 100% for submissions of post-tests as a necessary requirement for passing a course.

Mapping of students’ scores against their engagement levels will reveal students’ progress as well as their confidence with technical skills.

An increase in the number of departments that offer online modules and an increase in the percentage of the curriculum content that is provided as an online module is a positive indicator toward adopting the hybrid learning method.

Receiving standardized and global accreditation would be a seal of achievement. Implementation of our model in other universities, following our guidance and recommendations, would denote the influence of our program (Table 2).

**Table 2:** Summary of results from SOAR analysis.

**Strengths**	**Opportunities**
Regular update of knowledge	Clinical Skills training
Innovative learning	Time saving
Availability	Flexibility
Knowledge Sharing	Creativity
Abundance of information	Increased engagement
Accessibility	Standardized content
Source of Knowledge	Capacity building for students
Creativity	Capacity building for faculty
Increased engagement	Skills training
	Self-assessment
**Aspirations**	**Results**
Dedicated IT personnel	Achievement of periodic implementation of feedback
Introduce download option	Target 90% satisfaction rate among students/faculty
Panel of educational experts for periodic content review	Target 85% completion of e-modules
Increase students’ engagement (illustrative videos, 3D images, interactive flash games, etc)	Target 100% submissions of post-tests
Hire a digital designer to work with educational experts	Map students’ scores with their engagement levels, confidence with IT skills, cognitive levels, learning style, etc.
Standardize the e-learning process across universities	Increase in the department participation
Provide official faculty development program on IT and multimedia	Increase in curriculum delivery by hybrid learning method.
"Hours spent” metric and progress bar	Aim for standardization/global accreditation.
Interactive games and visuals for clinical skills development	
Forum discussion, post-test and self-assessment tests with personal leaderboard display	
Streamline student enrollment	
E-modules marks to be part of formative/summative evaluation	
Continuous development: periodic student/faculty feedback on content, technology, delivery, accessibility, quality, and other aspects that assess the quality of the e-learning experience.	

### Barriers

The four major themes that emerged under barriers and challenges to e-learning among undergraduate medical students in India were *eye strain*, *distractions*, *preference for conventional methodologies*, *and internet connectivity*. Online e-modules naturally increase the screen time spent in front of computers and mobile phones with the potential of leading to an increase in eye strain and other eye-related disorders. Distractions associated with e-modules include non-academic activities like playing games and social networking. Some students prefer conventional methods of learning such as using books and classroom lectures that allowed for the ‘human element’ to be present in learning. Lastly, although India is one of the biggest data consumers in the world, internet accessibility still remains limited especially in rural parts of the country. Other barriers identified in the study include the need for more creative e-learning delivery (going beyond PowerPoint presentations), the threat to physical fitness associated with having online sessions at home, and low computer literacy among students.

## Discussion

This study aimed at investigating the perceptions of undergraduate medical and dental students regarding e-learning / e-modules, and construct SOAR analysis based on the data collected. Administration of validated questionnaires based on the Knowledge, Attitude and Practices (KAP) model and the feedback questionnaire (FBQ) on e-modules allowed for access to students’ opinion regarding the use of e-modules in medical education. Around two-thirds of the students preferred blended learning (BL), a combination of e-learning (EL) and face-to-face (FTF) teaching, over absolute EL or FTF. They also held the view that EL should be viewed only as a supplement for traditional FTF teaching and not as a potential replacement for FTF.

Many students perceived FTF teaching as providing an opportunity to interact with teachers, friends, and support staff. They felt that real-time verbal feedback was more meaningful and personal compared to digital feedback during EL. FTF also provides the opportunity for students to learn professionalism from observing their peers and teachers. However, FTF teaching was reported to be associated with limited teacher interaction during lecture classes. To overcome this challenge, some students recommended the use of smart boards and interactive digital tools during regular lectures, and interactive quizzes at the end of each lecture to keep students motivated and engaged.

Some students felt that e-learning saved them time, allowing them to spend more time on other life activities. Muthuprasad (2021) documented a similar finding that many students prefer e-learning because of its flexibility [25]. Some participants indicated that e-modules helped them have more control over their learning process and also facilitated self-directed learning. This perception is in alignment with the findings of Paechter & Maier (2010) [28]. Some students identified the distribution of well-structured and standardized content to all learners as one of the main strengths of e-learning. The role of e-learning in facilitating the acquisition of appropriate competencies was also identified. Similar findings are supported by other authors as well [28, 29].

Conversely, some students identified eye strain, lack of human interaction, fall in motivation among students, low technology skills among faculty, coupled with non-engaging e-modules, as part of the current challenges for e-learning [28, 30]. Addressing these barriers will lead to enhanced student motivation and enhanced e-learning implementation in the institution.

During this pandemic, many higher education institutions for health professional education embraced e-learning to ensure continuity of teaching [31–33]. However, the persistence of the pandemic has created a need for sustainable policies and pedagogical practices [34, 35]. Some authors agree with the view that FTF sessions should be supplemented by a mix of asynchronous and synchronous online lessons which are content-rich and engaging [31, 32]. It is, therefore, very likely that higher education during the post COVID era will be in a blended mode with the combination of FTF and online sessions [30, 36].

The concept of readiness is important when designing, implementing, or assessing e-learning programs. Use of the KAP and FBQ aligned with determination of key attitudes that define readiness for e-learning in the literature. Organizational readiness refers to mental and physical preparation by the institution itself while readiness for e-learning among the medical students is based on the identification of predictors and barriers to e-learning [37–39]. Models of e-learning readiness in the literature identify two sets of variables: e-learning readiness variables (material and technological readiness, attitudinal readiness, mental health readiness, culture readiness) and inferential e-learning readiness [38, 39]. The findings from this study aligned with the e-learning readiness variables of material and technological readiness and attitudinal readiness, and inferential e-learning readiness.

While the focus is on students’ knowledge, attitude, and practice in regard to e-learning, faculty as individuals and teams collectively contribute to the success of any educational organization [20, 36, 37]. Hence, in addition to investing in digital infrastructure, the faculty has to be empowered to deliver blended learning (BL) through faculty development programs (FDP) [37]. Furthermore, continuous faculty development is a requisite for successful e-learning delivery [30, 36]. Systematic planning coupled with streamlined implementation of e-learning strategies will improve the dependability of training delivery by the institution [37]. SOAR analysis is ideal for achieving this objective.

As a dialogue-based system, SOAR leverages generative dialogue, declarative statements, and opinions generated from the KAP and FBQ questionnaires to create a strategic plan for e-learning in the research setting. Strengths in the context of this study refer to optimal use of e-learning and e-modules in medical education as well as capacities and assets that allow optimal performance [18, 20]; opportunities refer to situations and factors that may drive gains in e-learning for students such as cross-disciplinary collaborations [20, 40]; aspirations are visions and a long-term strategy that emerge from organizational strengths and capabilities, and results are outcomes from the execution of the strategy on e-learning and e-module [40]. An action plan for the use of e-module at the research setting emerged from the SOAR analysis.

There are four levels of activities in the action plan—infrastructural, student, faculty/institutional, and field levels. At the infrastructural level, the activities to be implemented are the creation of dedicated IT personnel and additions to technology infrastructure such as the introduction of a download option and various online educational tools identified to promote student learning.

At the institutional level, the activities to be implemented are creation of a panel of educational experts for periodic content review; engagement of a digital designer to work with educational experts; streamlining of student enrollment; and the implementation of continuous development programs (periodic student/faculty feedback on content, technology, delivery, accessibility, quality, and other aspects that assess the quality of the e-learning experience.

At the student level the activities to be implemented are increase students’ engagement through the use of strategies such as illustrative videos, 3D images, interactive flash games, etc.; introduction of "Hours spent” metric and progress bar; introduction of interactive games and visuals for clinical skills development; implementation of forum discussion, post-test and self-assessment tests with personal leaderboard display, and e-modules marks to be part of formative/summative evaluation. The field level activities are standardization of the e-learning process across universities and the provision of official faculty development program on IT and multimedia.

The results from the SOAR analysis provide targets and metrics for evaluating these activities. Targets and progress indicators will be developed to align with each activity item. Targets and indicators for institutional level activities include periodic implementation of feedback, achieving increase in the department participation, and increase in curriculum delivery by hybrid learning method. Indicators for student-level activities include target of 90% satisfaction rate among students/faculty; target of 85% completion of e-modules; target of 100% submissions of post-tests; and results from mapping of students’ scores with their engagement levels, confidence with IT skills, cognitive levels, learning style, etc. Targets for field-level activities include execution of progressive steps towards program standardization/global accreditation for e-learning in medical education and implementation of training programs for educators on e-learning.

The findings from this qualitative study are based on responses received from medical college students in a Private University in Chennai, India. The strengths of the study included the representative nature of the sample involving both genders across different cultures and socioeconomic status, and the study was conducted over a period of three years with three cohorts of students. The study was also conducted by Health Professionals who engage in both e-learning delivery and in medical education broadly. The limitations of the study include the small sample size (690) when compared to the size of the medical student population in India, the use of self-reported questionnaires (responses may be subject to personal bias), the use of a single institution limiting the generalizability of findings, ’hours spent’ metric as mentioned in the study may not be a good indicator of learning. This study did not consider the views of the faculty, technical team, and leadership. Present study did not compare the scores after the introduction of e-learning modules, and therefore could not establish the correlation / association between e-learning and academic performance. Some of the important limitations of E-Learning that were not addressed in the study include ‘limited opportunity to learn professionalism, need for academic integrity / self-discipline, endless distractions (home, workplace, online games, social media), inability to concentrate in isolation for long hours, superficial learning, and authenticity of the E-content’ [41].

## Conclusion

Development of structured and interactive e-modules in BL favors more engagement during learning and facilitates SDL. Implementation and management of BL with e-modules as an integral part of curriculum planning will promote CBME. A core team comprising of trained faculty, subject matter experts, qualified IT technicians, student representatives, secretaries, support staff, curriculum planners, assessment coordinators, quality assurance specialists, and educationists, will be necessary in order to achieve this goal. The findings from this SOAR analysis on the knowledge, attitude, practice, and feedback from a large sample of undergraduate medical and dental students may serve as a guide for institutions as they make strategic choices regarding BL.

### Recommendations

- Effective use of blended learning methodologies will deliver a combination of cognitive and psychomotor competencies to students.
- The ‘Flipped classroom’ approach can be considered for better outcomes for BL
- A core team of trained faculty, student volunteers, and qualified IT technicians may be crucial for development and validation of e-modules.
- Faculty development around e-learning technologies should be considered a high priority and receive prime attention.
- Online learning can be considered for formative assessment whereas FTF is ideal for summative assessments.
- Guidelines on ‘online etiquette’ for students and faculty should be made very clear during the beginning of the training program and emphasized throughout the program.
- It is advisable to have a stepwise strategic plan for establishment and sustainability of E-Learning infrastructure as it involves cost in terms of money, material and manpower.

## Supporting information

S1 Appendix The supporting data for this article is provided as a supplementary file. (DOCX)

## Abbreviations

BL: Blended learning
CBME: Competency-Based Medical Education
EL: E-learning
FBQ: Feedback questionnaire on e-modules
FTF: Face-to-Face learning
KAPQ: Knowledge, Attitude and Practice’ Questionnaire on e-learning
MCI/NMC: The Medical Council of India / National Medical Commission
OL: Online learning
SDL: Self-directed learning
SOAR analysis: ‘Strengths, Opportunities, Aspiration, Results’ analysis

## References

[1] Dhir SK, Verma D, Batta M, Mishra D. E-learning in medical education in India. Indian Pediatr. 2017;54(10):871–7. doi: 10.1007/s13312-017-1152-9 29120336

[2] Huynh R. The role of E-learning in medical education. Acad Med. 2017;92(4):430-. doi: 10.1097/ACM.0000000000001596 28350599

[3] Ellaway R, Masters K. AMEE Guide 32: e-Learning in medical education Part 1: Learning, teaching and assessment. Med Teach. 2008;30(5):455–73. doi: 10.1080/01421590802108331 18576185

[4] Ellaway R. E-learning: is the revolution over? Med Teach. 2011;33(4):297–302. doi: 10.3109/0142159X.2011.550968 21456987

[5] Harden RM. Ten key features of the future medical school—not an impossible dream. Med Teach. 2018;40(10):1010–5. doi: 10.1080/0142159X.2018.1498613 30326759

[6] World Health O. eLearning for undergraduate health professional education: a systematic review informing a radical transformation of health workforce development. 2015.

[7] Sofyan H, Anggereini E, Saadiah J. Development of E-Modules Based on Local Wisdom in Central Learning Model at Kindergartens in Jambi City. European Journal of Educational Research. 2019;8(4):1137–43.

[8] Kim K-J, Kim G. Development of e-learning in medical education: 10 years’ experience of Korean medical schools. Korean journal of medical education. 2019;31(3):205. doi: 10.3946/kjme.2019.131 31455050 PMC6715898

[9] Amandu GM, Muliira JK, Fronda DC. Using moodle e-learning platform to foster student self-directed learning: Experiences with utilization of the software in undergraduate nursing courses in a Middle Eastern university. Procedia-Social and Behavioral Sciences. 2013;93:677–83.

[10] Ibrahim NK, Al Raddadi R, AlDarmasi M, Al Ghamdi A, Gaddoury M, AlBar HM, et al. Medical students’ acceptance and perceptions of e-learning during the Covid-19 closure time in King Abdulaziz University, Jeddah. Journal of infection and public health. 2021;14(1):17–23. doi: 10.1016/j.jiph.2020.11.007 33341480 PMC7836241

[11] Dost S, Hossain A, Shehab M, Abdelwahed A, Al-Nusair L. Perceptions of medical students towards online teaching during the COVID-19 pandemic: a national cross-sectional survey of 2721 UK medical students. BMJ open. 2020;10(11):e042378. doi: 10.1136/bmjopen-2020-042378 33154063 PMC7646323

[12] Abbasi S, Ayoob T, Malik A, Memon SI. Perceptions of students regarding E-learning during Covid-19 at a private medical college. Pakistan journal of medical sciences. 2020;36(COVID19-S4):S57. doi: 10.12669/pjms.36.COVID19-S4.2766 32582315 PMC7306963

[13] Ananthakrishnan N. Competency based undergraduate curriculum for the Indian Medical Graduate, the new MCI curricular document: Positives and areas of concern. J Basic Clin Appl Health Sci. 2018;1:34–42.

[14] Sharma R, Bakshi H, Kumar P. Competency-based undergraduate curriculum: A critical view. Indian Journal of Community Medicine: Official Publication of Indian Association of Preventive & Social Medicine. 2019;44(2):77. doi: 10.4103/ijcm.IJCM_206_19 31333280 PMC6625257

[15] Bhandari B, Chopra D, Singh K. Self-directed learning: assessment of students’ abilities and their perspective. Adv Physiol Educ. 2020;44(3):383–6. doi: 10.1152/advan.00010.2020 32628525

[16] Morris TH, Rohs M. The potential for digital technology to support self-directed learning in formal education of children: A scoping review. Interactive learning environments. 2021:1–14.

[17] Talebian S, Mohammadi HM, Rezvanfar A. Information and communication technology (ICT) in higher education: advantages, disadvantages, conveniences and limitations of applying e-learning to agricultural students in Iran. Procedia-Social and Behavioral Sciences. 2014;152:300–5.

[18] Li L. The Management Implications of SWOT & SOAR Analysis of Classroom Dynamics: A Case Study in China. Organization Development Journal. 2020;38(4).

[19] Gürel E, Tat M. SWOT ANALYSIS: A THEORETICAL REVIEW Uluslararası Sosyal Araştırmalar Dergisi The Journal of International Social Research. Cilt; 2017.

[20] Stavros JM, Cole ML. SOARing towards positive transformation and change. Abac Odi Journal Vision Action Outcome. 2014;1(2).

[21] Omprakash A, Kumar AP, Sagar V, Sathiyasekaran BWC, Ramaswamy P. Needs Assessment Among Students of Health Professions Education for the Introduction of E-learning in a South Indian Tertiary Care University. Indian J Physiol Pharmacol. 2019.

[22] Sullivan GM, Artino AR Jr. Analyzing and interpreting data from Likert-type scales. J Grad Med Educ. 2013;5(4):541–2. doi: 10.4300/JGME-5-4-18 24454995 PMC3886444

[23] Almanasreh E, Moles R, Chen TF. Evaluation of methods used for estimating content validity. Research in social and administrative pharmacy. 2019;15(2):214–21. doi: 10.1016/j.sapharm.2018.03.066 29606610

[24] Braun V, Clarke V. APA handbooks in psychology^®^. APA handbook of research methods in psychology, Vol. 2. Research designs: Quantitative, qualitative, neuropsychological, and biological. American Psychological Association. 2012;57:71.

[25] Muthuprasad T, Aiswarya S, Aditya KS, Jha GK. Students’ perception and preference for online education in India during COVID-19 pandemic. Social Sciences & Humanities Open. 2021;3(1):100101. doi: 10.1016/j.ssaho.2020.100101 34173507 PMC7836920

[26] Visalam V, Kumar A, Prakash A, Padmavathi R. Knowledge, attitude and practice towards e–Learning among medical undergraduate students. IOSR J Appl Phys. 2015;7(4):1–4.

[27] Omprakash A, Kumar AP, Sagar V, Sathiyasekaran BW, Ramaswamy P. Medical Education/Original Article needs assessment among students of health professions education for the introduction of E-learning in a South Indian Tertiary Care University. Indian J Physiol Pharmacol. 2019;63(1):42–8.

[28] Paechter M, Maier B. Online or face-to-face? Students’ experiences and preferences in e-learning. The internet and higher education. 2010;13(4):292–7.

[29] Al-Azzam N, Elsalem L, Gombedza F. A cross-sectional study to determine factors affecting dental and medical students’ preference for virtual learning during the COVID-19 outbreak. Heliyon. 2020;6(12):e05704. doi: 10.1016/j.heliyon.2020.e05704 33324768 PMC7728427

[30] Aguilera-Hermida AP. College students’ use and acceptance of emergency online learning due to COVID-19. International Journal of Educational Research Open. 2020;1:100011. doi: 10.1016/j.ijedro.2020.100011 35059662 PMC7480788

[31] Amin HA, Shehata MHK, Ahmed SA. Step-by-step guide to create competency-based assignments as an alternative for traditional summative assessment. MedEdPublish. 2020;9. 10.15694/mep.2020.000120.1 PMC10702678 38073843

[32] Shehata MHK, Abouzeid E, Wasfy NF, Abdelaziz A, Wells RL, Ahmed SA. Medical education adaptations post COVID-19: an Egyptian reflection. Journal of Medical Education and Curricular Development. 2020;7:2382120520951819. doi: 10.1177/2382120520951819 32923673 PMC7457644

[33] Kumar AP, Al Ansari AM, Shehata MHK, Tayem YIY, Arekat MRK, Kamal AAM, et al. Evaluation of curricular adaptations using digital transformation in a medical school in Arabian gulf during the COVID-19 pandemic. Journal of Microscopy and Ultrastructure. 2020;8(4):186. doi: 10.4103/jmau.jmau_87_20 33623745 PMC7883498

[34] Macdougall C, Dangerfield P, Katz D, Strain WD. The impact of COVID-19 on Medical education and Medical Students. How and when can they return to placements? MedEdPublish. 2020;9(159):159. 10.15694/mep.2020.000159.1 PMC10702637 38073809

[35] Ehrlich H, McKenney M, Elkbuli A. Strategic planning and recommendations for healthcare workers during the COVID-19 pandemic. The American journal of emergency medicine. 2020;38(7):1446. doi: 10.1016/j.ajem.2020.03.057 32273142 PMC7194652

[36] Lapitan LDS Jr, Tiangco CE, Sumalinog DAG, Sabarillo NS, Diaz JM. An effective blended online teaching and learning strategy during the COVID-19 pandemic. Education for Chemical Engineers. 2021;35:116–31.

[37] Marinoni G, Van’t Land H, Jensen T. The impact of Covid-19 on higher education around the world. IAU global survey report. 2020;23.

[38] Yarime M, Tanaka Y. The issues and methodologies in sustainability assessment tools for higher education institutions: a review of recent trends and future challenges. Journal of Education for Sustainable development. 2012;6(1):63–77.

[39] Chakraborty M, Reddy YAK, Ghoshal JA, Amudharaj D, Tripathi M. Preparedness of medical students towards e-learning conducted during COVID-19 lockdown: A cross-sectional descriptive study. Journal of Education and Health Promotion. 2021;10. doi: 10.4103/jehp.jehp_1125_20 34667802 PMC8459856

[40] Cole ML, Stavros JM, Cox J, Stavros A. Measuring Strengths, Opportunities, Aspirations, and Results: Psychometric Properties of the 12-Item SOAR Scale. Front Psychol. 2022;13:1322. doi: 10.3389/fpsyg.2022.854406 35465545 PMC9028961

[41] Mukhtar K, Javed K, Arooj M, Sethi A. Advantages, Limitations and Recommendations for online learning during COVID-19 pandemic era. Pakistan journal of medical sciences. 2020;36(COVID19-S4):S27. doi: 10.12669/pjms.36.COVID19-S4.2785 32582310 PMC7306967

