# Supplementary material for: E-learning and E-modules in medical education—A SOAR analysis using perception of undergraduate students
Source: PLoS One. 2023 May 19;18(5):e0284882. doi: 10.1371/journal.pone.0284882 (PMC10198563; doi:10.1371/journal.pone.0284882)
Supplement: S1 Appendix — (DOCX) [file pone.0284882.s001.docx]

| ID | What one thing you **liked the most** about the e-module presentation | What one thing you **did not like** about the e -module presentation | What could have been done **better** in the e - module presentation | What are your **suggestions** for the improvement of the e –module presentation | **Other comments** |
| --- | --- | --- | --- | --- | --- |
| SDU059 | I was able to grasp knowledge in a well manner | Bit fast | Good overall | Some of the topics were skipped |  |
| SDU060 |  |  |  | If not done the next time, it would turn out more productive |  |
| SDU062 |  |  |  | More information about clinical side |  |
| SDU063 | Clear information about insulin | No comment | Can include more pictorial things |  |  |
| SDU064 | Animation and the fact it was integrated | It did not work, stopped working at some places | Questionnaires could have time limit | Could be much more simple in the way it is presented | I was very easy to learn from the e - module |
| SDU065 | How clearly it was explained | Too much time | Everything was understood well |  |  |
| SDU066 | To the point | There are hopes of betterment | Include more animations |  |  |
| SDU067 | It was interesting | It was too long |  | none |  |
| SDU068 | Explanations, mad easy to understand |  |  | More e- module presentations to be posted |  |
| SDU069 | Group discussion | pretest |  |  |  |
| SDU070 | Topics covered, time allotted was sufficient | No animations | More animations would make the e-module interesting |  |  |
| SDU073 | Animations, with audiovisual learning | Didn’t get information on from when to take assessment this time | More clear audio for few slides |  |  |
| SDU074 | Information was adequate |  | More images, a few videos |  |  |
| SDU075 | Topic chosen which i have not even seen | No comment | We want more class | Everything is good | Its really useful to me , Now I really know about diabetes mellitus, it is useful for my exam |
| SDU076 | Clear & animation was helpful |  | More animations | More animation with little more information will help us a lot |  |
| SDU077 | Every concept was clearly put & explained |  |  | I would like it if more topics are taught using e-module |  |
| SDU079 | Way of learning |  | No comments |  |  |
| SDU080 | Animated way of teaching, we understand more by seeing videos and photos, of what is actually happening | e- module is not working forsome people like me, I like the concept of e-module. | Everything is good | Fix this bug, otherwise everything is working good. | No comments |
| SDU081 | Peer group discussion | Everything was good | Till more content can be added | Its a good effort, hope it goes on well |  |
| SDU082 | interesting | nothing | More slides could have been added | Majority of the classes can be transformed into e-modules. | Regular classes can be converted like this, more topic could be added. |
| SDU083 | informative |  | voice |  |  |
| SDU084 | Easy learning | long class | Shorter class | No comments |  |
| SDU085 | It was colourful, had pictures | Bigger font | Bigger font | Put Bigger font |  |
| SDU086 | Simple statements | Pictures and 3D model | It was good the way it is | No comments |  |
| SDU087 | Clear & informative | No comments | More animations needed |  |  |
| SDU088 |  |  |  | It should be an interactive session |  |
| SDU089 | Easily understandable, especially with the animation of release of insulin | Recorded voic that playe was shrill |  | It would be better if it could be played in all the plugin & not just the flash | Easy access would be nice |
| SDU090 | Easy access,Techno saving(if accessible) | It didn’t work for me | Clarity of the audio could have been better | Provide references and use better font & quality animation |  |
| SDU091 | Interesting to know a lot | About the color & size of the font | More information in detail | No comments |  |
| SDU092 | Simple and brief |  | The voice synchronizationand format of presentation |  |  |
| SDU094 | Very good, whole lecture, | No comments , I liked it all | More cases with different situation | It is already very good & interesting |  |
| SDU095 | Pre & post test |  | More animation |  |  |
| SDU096 | Images a& audio |  |  | Some more audio |  |
| SDU098 | Précised way of teaching | Group discussions | nothing | nothing |  |
| SDU099 | Very attractive |  | Access even now is very difficult | Access | Great job, thank u all for your great effort |
| SDU100 | Simplicity of information given, making it easy to understand | Not much of visuals | Add more visuals( as in pictures, videos etc...) |  |  |
| SMU002 | presentation | 3 hrs class- too tiring | Consume less time, nothing |  |  |
| SMU009 | Very easy to study |  | Very short and able to understand easily |  |  |
| SMU015 | Very organised and content was displayed in a well manner with animations | Nothing specific , I could not access e- module at home as it had many links in slides |  | Key can be provided for those diagrams, but it is not very important as we can understand |  |
| SMU021 | Visual animations |  | More videos | More videos can be uploaded, necessary flow chart and diagrams can be put up |  |
|  |  |  |  |  |  |
| SMU024 |  | 3 hrs is too long | No, ur too good |  |  |
| SMU026 | I like the pre test & post test | Nothing | Everything was nice and it encouraged us for studying here its easy | It can be extended throughout this year |  |
| SMU027 | Animation was very good |  | Font and size of animation |  |  |
| SMU030 |  |  |  |  |  |
| SMU032 | Group discussion | Lecture font was not upto the mark | No comments | Can improve video quality |  |
| SMU037 | Interactive learning |  | Overall good |  |  |
| SMU039 | Audio & video were really so interesting, provoked interest | Nothing of that sort | Adequate time was not given to understand each slideclearly | It was heart touching, no other comments and suggestions | Adequate time could have been given to understand each slide clearly. Hope this will be very useful, the method of post test is very good, all the other subjects can also follow this kind of e-module. Animated slides are as such n mind so that students will never forget. I really liked it to the core. |
| SMU040 | Animations & visuals | Audio clarity and lack of information | More pictures and more information | Improved audio and more relevant information |  |
| SMU042 | Very colourful & interactive session | Time consuming | Students can be given back corrected worksheets to learn from previous mistakes | Booklets for each session can be given for revision of the particular session which will be very usefulfor future references |  |
| SMU061 | Group discussion & |  |  | No comments, but would like to have more e-modules in the future |  |
| SMU062 | post test |  |  |  |  |
| SMU063 | The teacher gave us the best knowledge | nothing | More animation |  |  |
| SMU065 | I liked the format | The length of it | The content could have been presented better | Needs to be more engaging |  |
| SMU068 | MCQ’S | It was quiet lengthy, after sometime was not able to listen, can reduce lecture. | Animations can be more | Animations can be more& slides can be less | It is an effective idea, the last e- module, JG apparatus was useful when I read it in book, It makes it more easier with good understanding |
| SMU069 | Animations which allowed clear understanding |  |  |  | One of the best Physiology lectures, left the lecture hall with clear understanding of the topic |
| SMU072 | The whole idea & pre & post test |  | It was well presented | This is fun way of learning |  |
| SMU073 | Animated clippings | No comments | Realistic approach | Full length discussion of a case | No comments |
| SMU074 | Enough time was given for understandingthe concepts, appropriate images and data was put up | nothing like that | More of case histories can be shown | The program can be conducted very often |  |
| SMU076 | Easily understandable | Its sufficient, I liked it | More & more animations | Information enough |  |
| SMU077 | Animation | The time spacingand voice some what more clear | Continuous animation, voice | No comments |  |
| SMU084 | presentation | format | Nothing much its good | Time is not adequate |  |
| SMU085 | Suited my style of learning, as it was in my school | Color & font | Size and color of font & slides | Improvement in the appearance of slides and the voice |  |
| SMU088 | Small group discussion following the lecture | It was little too long | More animations ought to have been added | Here teaching through small – group discussions |  |
| SMU090 | It was animated and informative | Time consuming | Time management | Post test is useful |  |
| SMU091 | Animated video along with explanation |  | More animations could be added |  |  |
| SMU095 | The concept of e- module is very innovative |  | More engaging activities to provoke further interest, like meeting a real patient with DM to understand its complications |  |  |
| SMU100 | Visual animation with video |  | The presentation with bigger font size& time for learning the slide | It is better if there are e- modules for anatomy and Biochemistry |  |
| SMU101 | The method pre & post test |  | The power point in website should be of downloadable form | Downloading the presentation from website | Even for anatomy and Biochemistry, we would prefer |
| SMU108 | Animations |  | I think there could have been more animations | It was good overall |  |
| SMU111 | Easy to understand and read | nothing | More video | More class we need |  |
| SMU118 | everything | nothing | Nee of many audios | Very interesting, kindled my interest, please follow it |  |
| SMU120 | It was very informative | There was less animation | There would have been more animations | If more animations, then easy to understand |  |
| SMU122 | About group discussion after the class | Less time of group discussion | Dividing people into small groups | Divide people into smaller groups | It was awesome |
| SMU124 | presentation | The ambience & the way it was taught. | The animation and the clinical aspects |  |  |
| SMU125 | I liked the animation video because, more understanding | nothing | More attractive with pictures & videos | Please put more pictures and videos for better understanding |  |
| SMU126 | Animated slide | Bit sleepy | Made interactive | Make it more interaction |  |
| SMU128 | simplicity | Lack of more written content | A separate theory column with diagrams | Same as above |  |
| SMU129 | Informative, clear, concepts explaine very well |  | More discussion time among peers |  |  |
| SMU133 | Visual animation |  | More videos |  |  |
| SMU143 | I was able to visually see how things hapened | Could have been more animation | More content would have been ok | More color,as the visuals were a bit dry, post test is a good idea. |  |
| SMU147 | Clear understanding of the concepts with more information | e-module having more problems while opening, troubleshooting the website is needed | More about drugs to treat DM | It was good , troubleshooting the website is needed. Pre and post test questions should be changed sothat you can assesswhether student has really understood. |  |
| SMU149 | Learning the things which I don’t know | nothing | No comments | No comments |  |
| SMU151 | It was interactive | Nothing as such | More explanation | It should be more interactive |  |
| SMU153 | Animations | To increase the speed of the session | More animations | The class was good |  |
| SMU158 | Different approach to learning | Very long class | More animations |  |  |
| SMU162 | Video animations were really good | - | - | Prior information and correction of problems in the website |  |
| SMU163 | A | A | A | A |  |
| SMU165 | It was interactive | The content was boring | There could be more animations | The animations could be better | Nice job |
| SMU167 | The animations about the angiotensin function was awesome | I would have loved if more animations were there | If there was a quiz round it would have been more fun and interesting | Quiz round, more animations | If all the lectures were given and post test forms 90 % of the students would be attentive in the class. Thanks for the session. |
| SMU170 | Informative | Lagged on for too long | Made more interesting |  |  |
| SMU172 | Presentation was apt for learning | No comments | More videos could be added | No comments |  |
| SMU173 | Different approach to learning | Sound quality was bad | - | No comments |  |
| SMU174 | Images and animations added were useful | Time consuming | It was perfect | No suggestions |  |
| SMU175 | The learning press with the help of slides | - | Technical setup |  |  |
| SMU176 | A |  |  |  |  |
| SMU177 | Videos animations were good |  | More animations | Increase animations |  |
| SMU178 | The ability to learn the course material summarily beforehand | Inaccessibility of the material | Better technical setup |  |  |
| SMU179 | It was very informative and interactive | Everything was very good and appropriate | It could not be more interactive | Audio was not working in Eli website |  |
| SMU190 | Mams way of explaining the things clearly and also the animations |  |  |  | It would be nice if every chapter were covered in this manner |
| SMU198 | No governance, free to do things at your own pace | No clarification by teachers/professors at home | Login audio issues could be addressed | Fix website issue, add a help line | N/A |
| SMU200 | Flexibility of time in which we can view | The non human way of teaching | Full screen mode | It could be more interactive |  |
| SMU201 | A |  |  |  |  |
| SMU202 | Animations and audio were good | We can’t ask doubts with teachers at home | Audio was not working, so try to fix it | Fix website login issues |  |
| SMU203 | Picture is good | No idea | More information | Audio is not played |  |
| SMU213 | Animations | There is no interaction |  |  |  |
| SMU214 | It was more clear with animations and audioversions | There were more technical difficulties, there was no audio in the e- module | Foe now it was upto expectations | No suggestions |  |
| SMU220 | Audio explanation and animations | no interaction | More visual explation of pictures and videos |  |  |
| SMU223 | Audio explanation which made me more curious about the topic | not interactive | To make the session short and precise |  |  |
| SMU224 |  |  |  |  |  |
| SMU225 | Audio | not interactive, no small group discussion | interaction | Interaction should be enhanced |  |
| SMU230 | Animations and flow charts used to explain the topic | Lack of interaction and discussion | More involvement of students | More interactive sessions |  |
| SMU238 | Specific and Apt , to the point, | Unable to concentrate on e- module and lethargic | Interactive with new technologic aspect |  |  |
| SMU242 | More attractive beause of post test | Sometimes becomes difficult to access |  |  |  |
| SMU246 | Both diagrammatic and audio created effective learning and even the self assessment | Could not see the animations | Posting the animation slides properly |  |  |
| SMU250 |  |  |  |  |  |
| SRD001 | The animations and audio clipping | I could not access to the e-module appropriately | Content should be improved , I think more content should be there. So that we can easily prepare for exams |  |  |
| SRD002 | The session was very much interesting and interactive | some technical errors | More videos can be uploaded | The session can be more interactive |  |
| SRD003 | The interactive class | nil | The audio would have been better |  |  |
| SRD006 | The focused atmosphere | nil | The audio would have been better | Add more visuals |  |
| SRD011 | The presentation was done with audio |  |  |  |  |
| SRD012 | The animations and interactive assignments | Could not access before class due to technical dificulty |  |  |  |
| SRD015 | PPT presentation was fine | The audio was not clear | Audio can be improved |  | good |
| SRD022 | Will develop learning | Takes a lot of time | No comments | No comments |  |
| SRD025 | It was interactive , slow and hence helped in easy understanding |  | More videos and animations can be used | More videos could be added |  |
| SRD028 | Understood easily | Nothing that I knew of | Nothing that I knew of, it was perfect | Awesome, more classes required |  |
| SRD029 | The e- module was to the point and all the objectives were met | There were a few technical difficulties | nil | A few more videos can be uploaded | nil |
| SRD034 | The interactive session | nil | The audio would have been better |  |  |
| SRD039 | The interactive session | There were many technical errors in it |  |  |  |
| SRD040 | More interesting and grabbed more attention | nothing | May be the time managing of a class | nothing | No comments |
| SRD041 | Innovative method | Technical difficulties | More organised | We would like more of such modules |  |
| SRD043 | interactive |  |  |  |  |
| SRD045 | Not mch | Time devouring | Better communication/ information convey | Not sure |  |
| SRD050 | Interactive session | test | No comments | No comments | No comments |
| SRD055 | This method was More interactive | We were not informed about the pretest. Not knowing the answer made me feel bad | Everything was good so far | Keep us informed beforehand about the topic to be discussed | As a hosteller , I am not able to have proper access to internet. It will be good if we get some reliable source of internet in our hostel. |
| SRD057 | The idea behind this, because of such classes. We atleast have some idea about the topic and we read something and came | For me it is working perfectly fine. For some the site or the presentation is not working properly. Just fix that, otherwise everything is good. | Little more of photos and animations, will make us understand better, we will get interested to read something. | No such suggestions. just wanted to highlight the above points | No comments |
| SRD058 | The audio learning was very useful & interesting | None, it was interesting | No idea | The group discussion can be little more enhanced | The E- module (especially the flip class e- module )was very effective. But I faced some problems initially in logging in. The contents were very brief |
| SRD060 | slides | pretest | Animations | More animated slides |  |
| SRD061 | The method of teaching | Internet assessment | It could have been a little short |  |  |
| SRD062 | It was interesting | Audio volume was very low | Audio volume could be increased | More animations could be included |  |
| SRD068 | Interactive session | No comments | It would have been better if there are more animations | Everything that was presented was good |  |
| SRD070 | Presentation and the slider | None , all were good | Animations | More e- module classes |  |
| SRD071 | The diagrams given were clear and easy to understand | Audio volume is less | Audio volume could be increased | More animations could be included |  |
| SRD072 | interesting | It was quite slow | More interesting coloured pictures |  |  |
| SRD073 | Animation on this topic | Test | No comments | More animations |  |
| SRD077 | It was interactive, helped me learn and understand more on the topic |  |  | More animations |  |
| SRD078 | No comments | Too much noise | No comments |  |  |
| SRD079 | No comments |  | Better animated pictures |  |  |
| SRD080 | I liked everything |  |  |  |  |
| SRD081 | Images and animations | No comments | interactive | No comments |  |
| SRD084 | It was new | e- module needs enrolment | Available without a login account | e- module to work in house |  |
| SRD088 | The voice was clear, simple, and points were easy to learn | Internet connection is week, the class was to long | Could have been shorter, with more descriptive pictures and flow charts | Start the e-learning in the form of mobile app. Please | Please start an app for this e- learning. The e-module sessions can be implemented in other subjects too |
| SRD092 | understandable | It was not upto the point | Animation |  |  |
| SRD093 | Relevant pictures and animation |  |  |  |  |
| SRD097 | interesting | No diagrammatic explanation through video |  |  |  |
| SRD100 | Asessment |  |  | No comments , everything was good |  |
| SRU001 | Viewing the slide show before the session online helps in reinforcing the concepts | Lack of internal connection prevents us from viewing the presentation before the class |  |  |  |
| SRU002 | It is easily accessible from anywhere | The class was for too long | Breaks in between sessions | Start an app for mobile phones |  |
| SRU003 | Lectures with audio |  | No comments | Interaction |  |
| SRU005 | The information was in small neat points |  |  |  |  |
| SRU010 | The points were understandable | The audio was not audible | More graphical representations | Flipped class e- module presentations can be made available on mobiles |  |
| SRU015 | Images and animations | Using PC | Instead of logging in at home please show it in the college |  |  |
| SRU018 | It is interesting to know the information presented in animation and videos | Ni comments | I wish the classes to be increased |  |  |
| SRU020 | The animation and the presentation | Time for answering the question is less |  |  |  |
| SRU022 | You can go home and review it | Internet connection is a problem. The class was too long | Short and more pictures | Start an App for phones |  |
| SRU036 | The concepts were presented | It was time consuming | Not so lengthy |  |  |
| SRU038 | Very interesting and interactive, provoked my interest. The contents were so good and we can easily understand | We can’t get the audio and not accessible in our phones | Some interaction with the students |  | Good interesting, easily understandable, provoked my interest |
| SRU040 | Very creative | Difficulty in accessing it |  |  |  |
| SRU041 | It was well structured and well timed | The absence of human element made it uninteresting and monotonous | If it was made more intersting | The oratory should be a little more enthusiastic |  |
| SRU042 | a test after it | Non concise | Could be less cumbersome |  |  |
| SRU046 | The way of teaching | Formative assessment | nothing | nothing |  |
| SRU049 | Animations made the session intersting | Concepts once told are not repeated again | No comments | Explaining the concepts again | No comments |
| SRU054 | Quizzing through explanation. It is different kind of learning, provoked interest, Applied aspect emphasized. | End of the day |  | post test to see self improvement. Online test made mandatory to complete |  |
| SRU055 | New and different | boring | More simplified , not much technical terms, more animations | Should be more informative |  |
| SRU056 | It was interesting |  |  |  |  |
| SRU058 | Creative animated pictures | No comments | No comments | No comments |  |
| SRU059 | The questions and the audio-video presentation | The audio was not proper, takes more time but time as it helps us to study in itself. | Including hand drawn diagrams renam points. |  | Good work, very helpful& interesting, & prefer for upcomimg classes, also to be taken for afternoon classes like this so we can utilise the time. |
| SRU061 | The explanation provided with the presentation | none | More pictorial presentation | Introduction of cases etc |  |
| SRU063 | Animated picture | No comments | Some more linked graph |  |  |
| SRU067 | Easier to understand layout | distraction | No coments | Allow tablets to be used so that each one can learn at their own pace. |  |
| SRU069 | The clear definitions that were provided for each process | The technical difficulties that we at the beginning | More diagrams would have been nice | Setting up the presentation prior to e- module to the same time |  |
| SRU075 | Pretest, I liked the most to recollect | No comments |  |  |  |
